# Lumbar Pedicle Morphometry in the Mexican Population: Defining Anatomical References for Safer Spine Surgery

**DOI:** 10.7759/cureus.91799

**Published:** 2025-09-07

**Authors:** Tomas Moncada-Habib, Tania Jiménez-Molina, Marco Antonio Muñuzuri-Camacho, Paula Andrea Pulido Bayona, Kevin S Toache, Jesús Álvaro Heredia, German López-Valencia, Juan Antonio Alvaro-Heredia

**Affiliations:** 1 Neurosurgery, Instituto Nacional de Neurología y Neurocirugía Manuel Velasco Suárez, Mexico City, MEX; 2 Neurosurgery, Hospital Universitario Infantil de San José, Fundación Universitaria de Ciencias de la Salud, Bogotá, COL; 3 Neurosurgery, Hospital de Especialidades, Centro Medico Nacional Siglo XXI, Mexico City, MEX; 4 Neurosurgery, Universidad de Guerrero, Guerrero, MEX; 5 Neurosurgery, Instituto Nacional de Neurología y Neurocirugía Manuel Velasco Suarez, Mexico City, MEX

**Keywords:** lumbar pedicle dimensions, mexican population, morphometry, pedicle screw fixation, vertebral body anatomy

## Abstract

Background: Pedicle screw fixation is a fundamental technique in spine surgery that requires precise knowledge of lumbar pedicle morphology to ensure safe and effective instrumentation. Pedicle dimension variations are influenced by sex, age, and ethnicity.

Materials and methods: This retrospective observational study analyzed 185 lumbar vertebrae (L1-L5) from the CT scans of 37 adult patients without structural spinal pathologies. Bilateral measurements of pedicle width, height, length, and transverse angle, as well as vertebral body length, width, and height, were obtained using digital measurement tools. Statistical comparisons were performed by sex and age, with significance determined using independent t-tests and Spearman’s correlations.

Results: The pedicle width increased from L1 (7.0 mm) to L5 (11.8 mm), whereas the pedicle height decreased in the caudal direction. The transverse pedicle angle widened from L1 (16.8°) to L5 (21.9°). Males exhibited significantly greater pedicle width and length than females (p < 0.01), whereas vertebral body dimensions showed no significant sex-based differences. Age correlated negatively with pedicle height and angle (Rho ≈ -0.27 to -0.30), suggesting anatomical remodeling with age. No correlation was found between age and the dimensions of the vertebral bodies.

Conclusions: This study is among the first to provide detailed lumbar morphometric data for a Latin American population, specifically the Mexican population. These findings align with global trends and highlight the importance of considering population-specific anatomical differences during surgical planning.

## Introduction

Pedicle screw fixation is a cornerstone of spinal surgery and is widely employed in the management of degenerative diseases, traumatic injuries, neoplastic lesions, and vertebral deformities. The accuracy of screw placement depends on a precise understanding of lumbar pedicle morphology, as deviations in orientation or size may compromise the neurovascular structures and jeopardize implant stability [1]. The dimensions of the lumbar pedicle vary systematically across vertebral levels, and factors such as sex, height, and body weight further influence these measurements [2]. Such variability underscores the need for individualized surgical planning tailored to each patient’s unique anatomy.

Previous research has demonstrated that lumbar pedicle morphology differs among populations, with ethnic background and sex being significant determinants [1,3-11]. Most of these findings were derived from tomographic reconstructions, emphasizing the importance of accounting for population-specific anatomical diversity when determining screw dimensions and trajectories during spinal surgery.

Furthermore, the advent of advanced techniques, such as cortical bone trajectory fixation, has heightened the demand for detailed three-dimensional analyses of the lumbar pedicle anatomy [12]. Despite the increasing availability of anatomical datasets, comprehensive morphometric descriptions of lumbar pedicles in Latin American populations remain scarce.

The primary objective of this study was to provide a detailed morphometric characterization of the lumbar pedicle in adult Mexican patients without structural spinal pathology and to establish population-specific reference values. As secondary objectives, we sought to describe vertebral body morphology across lumbar levels, analyze sex- and age-related variations, and discuss the clinical implications of these measurements for surgical planning, implant selection, and optimization of screw trajectories in spinal fixation.

## Materials and methods

Study design and population

This retrospective observational morphometric study analyzed lumbar spine CT scans of Mexican adult patients evaluated for non-pathological indications at a tertiary neurosurgical center. Thirty-seven patients met the inclusion criteria, each contributing measurements from all five lumbar vertebrae (L1-L5), resulting in a total of 185 evaluated vertebrae.

Inclusion and exclusion criteria

The inclusion criteria comprised adult patients (≥18 years) with complete high-resolution lumbar CT scans performed for non-pathological indications. The exclusion criteria were as follows: history or radiological evidence of spinal fractures, deformities, or congenital anomalies; spinal neoplasms, infections, or inflammatory conditions; previous lumbar instrumentation or surgery; or incomplete or poor-quality imaging that prevents accurate measurements.

Image acquisition and measurements

**Figure 1:**
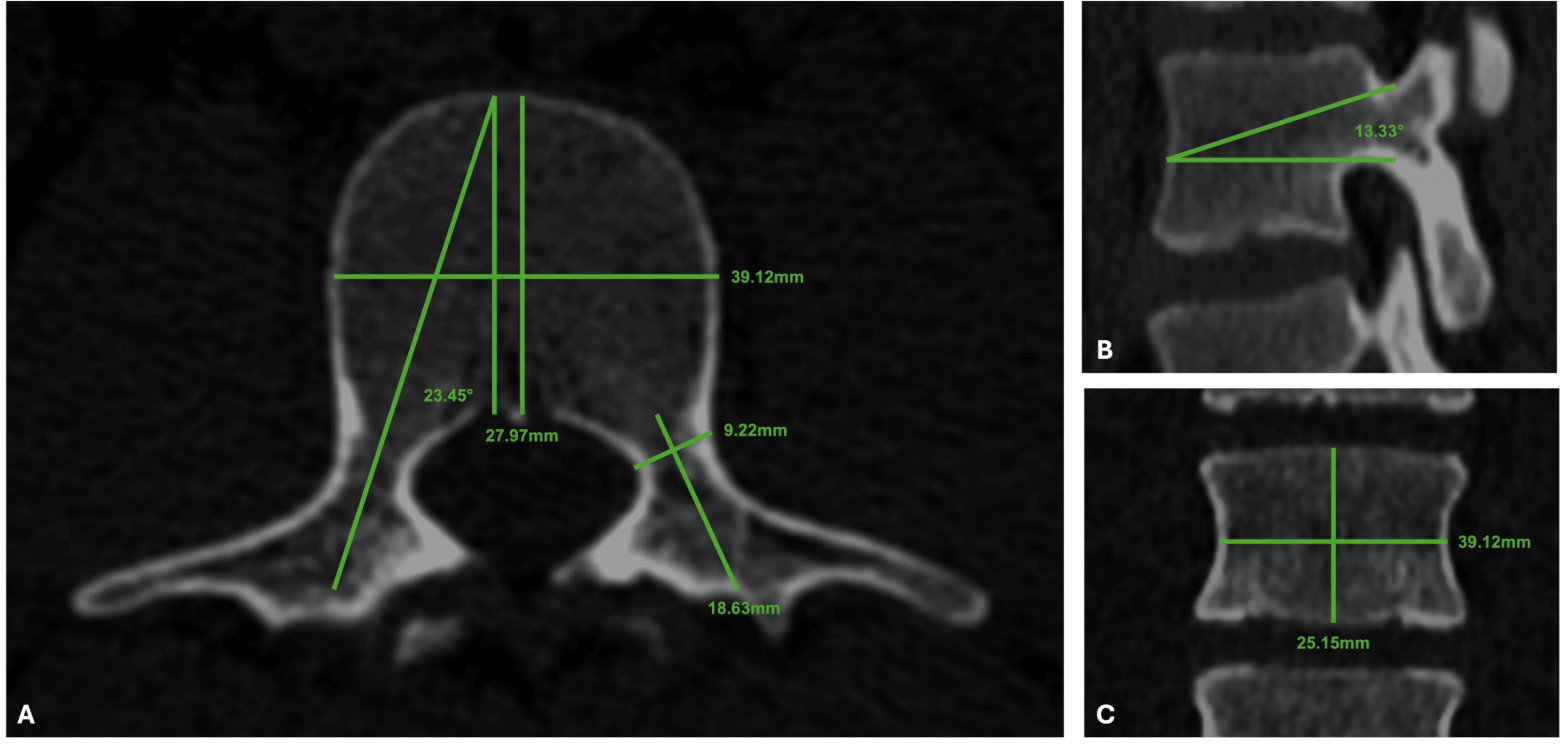
CT images of the L3 vertebra from an adult patient without structural spinal pathology Illustrating the digital measurements applied in this study. (A) Axial view showing the transverse pedicle angle (23.45°), pedicle width (9.22 mm), pedicle height (18.63 mm), pedicle length (27.97 mm), and vertebral body width (39.12 mm). (B) Sagittal view displaying the sagittal pedicle angle (13.83°). (C) Coronal view presenting vertebral body height (25.15 mm) and width (39.12 mm). These anatomical parameters were included in the morphometric analysis and served to calculate mean values and compare differences by sex and age within the evaluated Mexican population. CT: computed tomography

**Figure 2:**
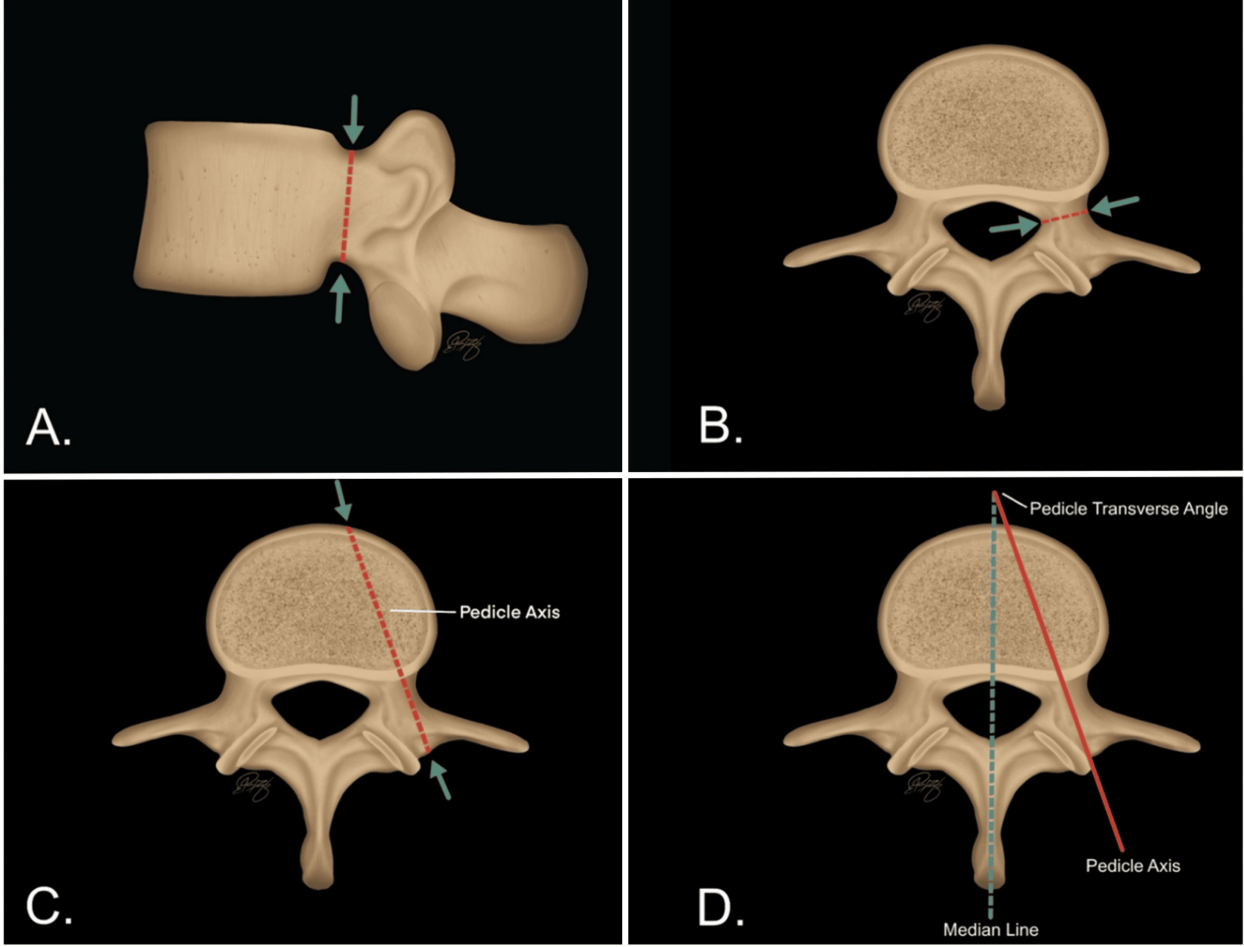
Schematic representation of the anatomical planes and axes used for pedicle measurements in lumbar vertebrae (A) Sagittal view showing the pedicle axis relative to the vertebral body. (B) Axial view demonstrating the orientation of the pedicle and entry point along the transverse plane. (C) Superior axial view identifying the pedicle axis along its longitudinal trajectory. (D) Measurement of the pedicle transverse angle, defined as the angle between the pedicle axis and the vertebral midline. These reference planes were used to guide digital measurement acquisition and ensure consistency in morphometric analysis. Image Credit: Paula A. Pulido-Bayona

Images were acquired with a slice thickness of 1.0 mm, a field of view of 250 mm, a tube voltage of 120 kVp, and a tube current between 200 and 250 mA. Multiplanar reconstructions were generated using a bone algorithm to optimize cortical definition. High-resolution axial, coronal, and sagittal CT images were reviewed using PACS workstations with digital calipers. The following anatomical parameters were measured bilaterally for each lumbar vertebra: pedicle height (PH), pedicle width (PW), pedicle length (PL), pedicle transverse angle (PA), corpus length (CL), corpus width (CW), anterior vertebral body height (AH), and posterior vertebral body height (PH) (Figures 1-2).

Measurements were performed independently by two observers trained in spinal anatomy and verified by a senior spine surgeon. Discrepancies greater than 2 mm or 2° were resolved by consensus with a third reviewer.

Data processing and statistical analysis

Data were recorded in Microsoft Excel (Microsoft Corp., Redmond, WA, USA) and analyzed using SPSS Statistics version 25 (IBM Corp., Released 2017. IBM SPSS Statistics for Windows, Version 25.0. Armonk, NY: IBM Corp.). Descriptive statistics, including the mean, standard deviation, minimum, maximum, and sample size, were calculated for each parameter at every vertebral level (L1-L5). Sex distribution and age range were also reported in the study. Sex-based comparisons were performed using independent-sample t-tests for each morphometric variable. Associations between age and anatomical parameters were assessed using Spearman’s correlation coefficients, including pedicle and vertebral body measurements.

Ethical considerations

The requirement for ethical approval was waived because of the retrospective design and anonymized nature of the dataset.

## Results

A total of 37 patients (54.1% men and 45.9% women) were included in the study. The mean age was 60.4 years (SD 15.8; range, 26-88). Each participant contributed morphometric data from five lumbar vertebrae (L1-L5), yielding a complete dataset for all vertebral levels.

Vertebral body measurements

As shown in Table 1, the vertebral body dimensions demonstrated progressive anatomical changes from L1 to L5. The AH increased from a mean of 24.3 mm at L1 to 26.2 mm at L5. The CL and CW also increased in the caudal direction, ranging from 29.4 mm and 40.3 mm at L1 to 32.4 mm and 49.5 mm at L5, respectively. In contrast, the PH decreased from 26.9 mm at L1 to 22.5 mm at L5, which was consistent with the lumbar curvature and load-bearing adaptations.

**Table 1:** Descriptive statistics of vertebral body dimensions (L1-L5) in 37 Mexican adults Values are presented as mean ± SD. AH: anterior vertebral body height, PH: posterior vertebral body height, CL: vertebral body length, CW: vertebral body width, SD: standard deviation

(n = 37)	Statistic	AH	CL	CW	PH
L1	Mean ± SD	243.1 ± 19.1	294.3 ± 31.9	402.6 ± 45.3	268.9 ± 18.3
	Min - max	200 - 248	228- 360	315 - 496	236 - 309
L2	Mean ± SD	251.6 ± 22.8	307.7 ± 34.3	421.6 ± 42.9	267.5 ± 19.5
	Min - max	212 - 316	249 - 408	286 - 485	227 - 295
L3	Mean ± SD	256.4 ± 16.7	316.6 ± 31.1	440.2 ± 45.0	261.1 ± 24.3
	Min - max	220 - 285	262 - 414	335 - 525	213 - 310
L4	Mean ± SD	253.8 ± 20.8	318 ± 26.8	455.2 ± 46.9	250 ± 24.5
	Min - max	206 - 290	261 - 368	350 - 537	205 - 319
L5	Mean ± SD	261.8 ± 27.8	324.3 ± 36.2	495.4 ± 56.8	224.9 ± 27.1
	Min - max	206 - 330	260 - 439	352 - 636	163 - 311

Pedicle morphometry

Table 2 summarizes the left and right pedicle dimensions for each lumbar level. The transverse pedicle angle increased steadily from L1 to L5 bilaterally, rising from 16.8° (left) and 16.7° (right) at L1 to 21.9° at L5. Left PH/right PH decreased caudally, from 12.8 mm/13.0 mm at L1 to 10.2 mm bilaterally at L5. The PL remained relatively constant, with a slight reduction toward L5 (40.4 mm to 37.8 mm on the left and 40.3 mm to 38.6 mm on the right). In contrast, the left PW/right PW increased notably from 7.0 mm / 6.7 mm at L1 to 11.8 mm / 12.4 mm at L5.

**Table 2:** Descriptive statistics of left and right pedicle dimensions (L1-L5) in 37 Mexican adults Values are presented as mean ± SD. LPW: left pedicle width, LPH: left pedicle height, LPL: left pedicle length, LPA: left pedicle transverse angle, RPW: right pedicle width, RPH: right pedicle height, RPL: right pedicle length, RPA: right pedicle transverse angle, SD: standard deviation

(n = 37)	Statistic	LPA	LPH	LPL	LPW	RPA	RPH	RPL	RPW
L1	Mean ± SD	16.8 ± 1.9	12.8 ± 1.4	40.4 ± 6.2	7.0 ± 1.7	16.7 ± 2.0	13.0 ± 1.5	40.3 ± 6.2	6.7 ± 1.6
	Min - max	11.7 - 21.2	11.0 - 17.0	20.0 - 49.0	4.0 - 10.7	11.2 - 21.0	9.0 - 16.0	20.0 - 48.0	4.0 - 9.2
L2	Mean ± SD	16.5 ± 2.3	12.3 ± 1.8	40.6 ± 6.5	7.0 ± 1.5	16.4 ± 2.0	12.5 ± 1.6	41.1 ± 6.5	7.0 ± 1.6
	Min - max	10.1 - 20.7	8.0 - 17.0	20.0 - 49.0	4.0 - 9.7	11.6 - 20.7	8.0 - 15.0	21.0 - 49.0	4.0 - 9.8
L3	Mean ± SD	17.0 ± 2.4	12.1 ± 1.8	41.3 ± 6.8	8.1 ± 1.8	17.0 ± 2.6	12.0 ± 1.9	41.3 ± 6.6	7.6 ± 2.0
	Min - max	10.8 - 21.6	8.0 - 16.0	21.0 - 51.0	3.8 - 11.0	11.0 - 21.2	8.0 - 16.5	21.0 - 50.0	3.9- 12.0
L4	Mean ± SD	18.6 ± 1.9	11.3 ± 1.6	40.5 ± 6.0	9.3 ± 1.8	18.6 ± 1.9	10.9 ± 1.5	40.5 ± 6.3	8.9 ± 1.9
	Min - max	15.5 - 23.0	9.0 - 15.0	20.0 - 48.0	5.5 - 13.0	14.7 - 23.0	8.14 - 14.0	20.0 - 48.0	5.3 - 12.0
L5	Mean ± SD	21.9 ± 2.8	10.2 ± 1.6	37.8 ± 5.5	11.8 ± 3.0	21.9 ± 2.5	10.2 ± 1.4	38.6 ± 6.0	12.4 ± 2.5
	Min - max	16.8 - 29.0	7.0 - 13.0	23.0 - 47.0	5.2 - 18.0	16.1 - 29.0	7.9 - 14.0	22.0 - 50.0	6.0 - 18.8

Sex-based anatomical variations

As detailed in Table 3, independent-samples t-tests revealed statistically significant sex-based differences in several pedicle parameters. Male patients consistently had larger PW and PL than female patients. For example, the right PW was greater in males (mean, 8.75 mm) than in females (mean, 7.65 mm; p = 0.005), and the right PL was longer in males (42.47 mm vs. 36.87 mm; p < 0.001). Similar differences were observed on the left side for width (8.75 vs. 7.65 mm; p ≈ 0.005) and length (42.47 vs. 36.87 mm; p < 0.001). Other variables, including PH and PA, did not reach statistical significance, although a consistent trend toward larger dimensions in men was observed. No significant sex-related differences were found in the vertebral body dimensions (CL, CW, AH, and PH).

**Table 3:** Sex-based comparison of lumbar pedicle and vertebral body dimensions Values are presented as mean ± standard deviation. *p < 0.05; **p < 0.01, independent-samples t-test. RPW: right pedicle width, RPH: right pedicle height, RPL: right pedicle length, RPA: right pedicle transverse angle, LPW: left pedicle width, LPH: left pedicle height, LPL: left pedicle length, LPA: left pedicle transverse angle, AH: anterior vertebral body height, PH: posterior vertebral body height, CL: vertebral body length, CW: vertebral body width

Variable	Female mean ± SD	Male mean ± SD	T-statistic	P-value
RPH	11.39 ± 1.83	11.83 ± 1.88	-1.67	0.097
RPW	7.65 ± 2.86	8.74 ± 2.66	-2.83	0.005
RPL	36.87 ± 6.62	42.46 ± 5.25	-6.26	0.000
RPA	18.10 ± 3.05	18.33 ± 2.96	-0.55	0.586
LPH	11.43 ± 1.75	11.9 ± 1.97	-1.71	0.088
LPW	7.76 ± 2.65	8.82 ± 2.56	-2.95	0.004
LPL	36.87 ± 6.65	42.10 ± 5.21	-5.80	0.000
LPA	17.94 ± 3.15	18.32 ± 2.91	-0.88	0.383
CL	292.58 ± 29.53	323.52 ± 29.81	-7.63	0.000
CW	416.65 ± 58.94	459.52 ± 47.68	-5.73	0.000
AH	248.23 ± 17.44	258 ± 24.70	-3.32	0.001
PH	251.21 ± 27.61	255.77 ± 27.86	-1.17	0.245

Age-related correlations

Spearman’s correlation analysis (Table 4) showed significant negative correlations between age and specific pedicle parameters. Age was inversely correlated with right pedicle height (Rho = -0.27, p = 0.0003), left pedicle height (Rho = -0.30, p < 0.0001), right pedicle transverse angle (Rho = -0.16, p = 0.034), and left pedicle transverse angle (Rho = -0.19, p = 0.010). No significant correlations were found between age and PW or PL, or between age and vertebral body measurements.

**Table 4:** Spearman’s correlation coefficients between age and lumbar morphometric parameters *p < 0.05; **p < 0.01 RPW: right pedicle width, RPH: right pedicle height, RPL: right pedicle length, RPA: right pedicle transverse angle, LPW: left pedicle width, LPH: left pedicle height, LPL: left pedicle length, LPA: left pedicle transverse angle

Variable	Spearman Rho	P-value
RPH	-0.27	0.000
RPW	-0.10	0.166
RPL	-0.12	0.092
RPA	-0.16	0.034
LPH	-0.30	0.000
LPW	-0.12	0.113
LPL	-0.17	0.020
LPA	-0.19	0.010

## Discussion

A detailed understanding of the lumbar spine pedicle anatomy is essential for safe and effective spinal surgery, particularly in procedures involving pedicle screw fixation. This study, conducted at the National Institute of Neurology and Neurosurgery in Mexico, represents one of the first comprehensive morphometric analyses of lumbar pedicles in Latin American populations. Our findings reinforce the principle that anatomical variability should guide surgical planning for individual patients and specific populations. When contextualized with global data from the past decade, these results provide critical insights for optimizing spine surgery outcomes.

In this cohort of 37 patients, the pedicle width nearly doubled from the cranial to the caudal lumbar levels, whereas the pedicle height decreased, a pattern consistent with global morphometric reports [7,9]. Specifically, the pedicle width increased from a mean of 7.0 mm at L1 to 11.8 mm at L5. Meanwhile, the height showed a declining trend, confirming similar findings in South Asian and East Asian populations [2,5]. These parameters are essential for selecting the appropriate screw diameter and minimizing the risk of neurovascular compromise.

The transverse pedicle angle, a key determinant of the screw trajectory, also increased caudally from 16.8° at L1 to 21.9° at L5. This finding mirrors previous studies, which emphasized the need for angulation adjustments at lower lumbar levels. Such consistency across ethnicities suggests a shared biomechanical adaptation to the increased load-bearing demands of the lower spine in both sexes [8,13].

Sex-based anatomical differences were particularly pronounced in the present study. Male patients exhibited significantly greater pedicle widths and lengths, consistent with morphometric data from Turkish, Indian, and New Zealand populations [7,8,14]. These differences underscore the risk of suboptimal screw fit when sex is not considered, potentially compromising the fixation strength or placing adjacent neurovascular structures at risk. Interestingly, vertebral body dimensions did not differ significantly between the sexes, suggesting a more conserved morphology than that of the pedicles. This highlights the greater relevance of pedicle-specific customization in surgical planning.

Age was inversely correlated with pedicle height and transverse angle (Rho = -0.27 to -0.30), consistent with age-related degenerative changes [1,12]. These morphological shifts may reflect remodeling and reduced bone quality with aging, factors that warrant careful consideration when planning pedicle screw trajectories in older patients to prevent complications such as screw loosening or cortical breach.

From a broader perspective, the need for population-specific data becomes clear when international studies are compared. Even within similar ethnic groups, pedicle morphology can vary due to factors such as body mass index, genetic background, and environmental influences [4,15]. This variability supports the conclusion that reliance on international reference values may not be sufficient for Latin American populations in general.

The use of CT-based three-dimensional morphometry in this study aligns with modern preoperative planning trends and complements advanced 3D segmentation and reconstruction techniques for precise anatomical assessment. Integrating such tools into surgical workflows can enhance screw placement accuracy, reduce operative time, and minimize intraoperative complications [16,17].

This study expands the global understanding of lumbar pedicle morphology by providing population-specific reference values for Mexican adults, revealing important sex- and age-related variations. Our findings align with international trends, underscoring the anatomical distinctions that may influence implant selection and screw trajectory planning. Incorporating these insights into neurosurgical practice can support more precise and personalized spinal surgery; however, their direct impact on surgical safety remains hypothesis-generating and should be validated in future prospective multicenter studies, including the potential integration of AI-assisted anatomical analyses in preoperative planning.

This study is limited by its relatively small sample size, which is a single center, and thus restricts generalizability. Measurement reproducibility may be affected since inter-observer reliability was not formally quantified, and practical applicability remains constrained without intraoperative validation. However, these limitations do not detract from the study's strengths and novelty; instead, they highlight the need for future multicenter investigations with larger cohorts to validate and expand upon these findings.

## Conclusions

A precise understanding of lumbar pedicle and vertebral body morphology is essential for safe spinal surgery, as it guides implant sizing and optimal screw trajectories and reduces complications such as cortical breach, loosening, or implant failure. Our study provides population-specific reference values for the Mexican cohort, highlighting significant sex- and age-related variations that have direct implications for preoperative planning and surgical safety. Future studies with larger, multicenter cohorts are warranted to validate these findings and further explore technological tools, such as AI-assisted morphometric analysis, to enhance surgical precision and efficiency.
